# Diverticulitis in the sigmoid colon presenting with only lower anterior chest pain

**DOI:** 10.1002/ccr3.3533

**Published:** 2020-11-18

**Authors:** Masaki Tago, Risa Hirata, Yoshio Hisata, Oie Satsuki, Tokushima Yoshinori, Hidetoshi Aihara, Motoshi Fujiwara, Shu‐ichi Yamashita

**Affiliations:** ^1^ Department of General Medicine Saga University Hospital Saga Japan

**Keywords:** chest pain, differential diagnosis, diverticulitis, referred pain

## Abstract

An 80‐year‐old man who presented with only lower anterior chest pain was diagnosed as sigmoid colon diverticulitis. His chest pain was considered to be referred pain from a disease at sigmoid colon, which should be suspected when other major causes of chest pain are excluded.

## Graphical abstract

Lower chest pain due to referred pain can rarely occur as the only symptom in patients with sigmoid colon diverticulitis. When evaluating patients with lower chest pain, try to examine abdomen carefully after ruling out major causes of chest pain.

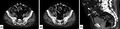

## 1 INTRODUCTION

The relations between sites of pain and the organs involved are complicated, partly because of the presence of referred pain, which may be felt in a part of the body that is distant from, and apparently irrelevant to, the causative organ. We previously reported diagnostically challenging cases in which the only presenting problem was referred pain, such as a case of acute cholecystitis with only lower chest pain ^1^ or one of acute aortic dissection with only left mandibular pain.^2^

Abdominal pain—usually felt in the left lower abdomen of Caucasians with a high incidence of sigmoid colon diverticula, or in the right or mid‐lower abdomen by Asians with a high incidence of right colon diverticula—is the most common symptom of diverticulitis.^3^, ^4^, ^5^ The pain might last several days.^6^ Colonic diverticulitis associated with chest pain alone (no abdominal pain) is so rare that we were unable to find any previously reported cases.

When seeing a patient with acute chest pain, it is mandatory, first, to exclude the “five deadly causes of chest pain” (ie, acute coronary syndrome, acute aortic dissection, pulmonary embolism, tension pneumothorax, pericardial tamponade ^7^). Nevertheless, referred pain from colonic disease should be suspected when each of the five, more‐serious, potentially fatal disorders have been eliminated.

## 2 CASE

An 80‐year‐old Japanese man was admitted to our hospital with a 6 hours history of lower anterior chest pain that had not been alleviated by sublingual nitroglycerin, dyspnea, and generalized perspiration. A few days previously, he had begun to experience a tight feeling in his lower anterior chest, which lessened with nitroglycerin administration. Over the years, he had been treated for aortic regurgitation, chronic heart failure, vasospastic angina, hypertension, hypercholesterolemia, and chronic obstructive pulmonary disease at an outpatient clinic established by his family physician. His past history included mitral valvuloplasty 12 years previously for mitral stenosis and regurgitation. He had smoked 50 cigarettes per day for 50 years until 12 years ago, and he was allergic to radiocontrast agents. On arrival at the hospital, he was alert and did not complain of abdominal pain. His body temperature was 36.0°C, blood pressure 135/76 mmHg, pulse rate 80 beats/min, respiration rate 26 breaths/min, and percutaneous oxygen saturation 96% while breathing room air. Physical examination revealed an apical systolic murmur, without tenderness or a skin rash at the site of the chest pain, and there was no leg edema. His abdomen was soft and flat, with the left lower portion tender without guarding.

**Figure 1:**
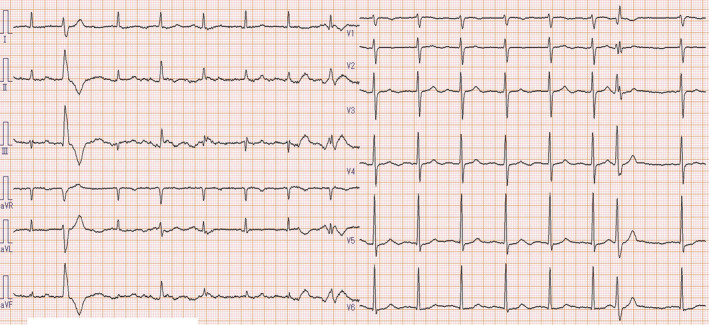
Admission electrocardiography shows ventricular premature contraction without findings that would indicate ischemia (eg, changes in ST‐T segments in comparison with previous records)

**Figure 2:**
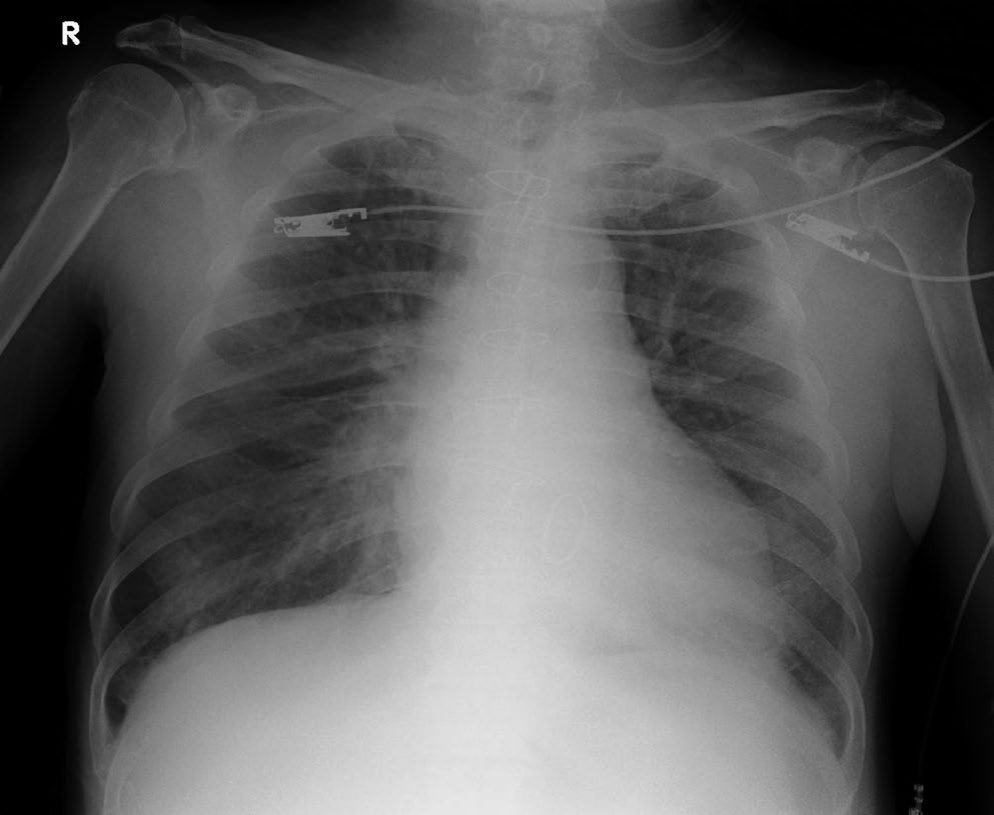
Admission chest radiography. Anteroposterior image reveals mild heart enlargement and slightly increased pulmonary vascular markings

Laboratory examination results are shown in Table 1. The blood cultures performed on his arrival were negative. The electrocardiogram showed normal sinus rhythm and ventricular premature contractions without relevant findings indicating cardiac ischemia, myocarditis, or pericarditis, such as changes in ST‐T segments in comparison with his previous records (Figure 1). Anteroposterior chest radiography revealed mild heart enlargement and slightly increased pulmonary vascular markings (Figure 2). Transthoracic echocardiography showed moderate mitral regurgitation without left ventricular asynergy. Although the right ventricular systolic pressure was as high as 51 mmHg, it was lower than that examined previously.

**Table 1:** Laboratory findings at the time of the patient's admission

Complete blood cell counts				T‐bil	1.3	mg/dL
WBC	5.8 × 10^3^		/μL	γ‐GTP	258	U/L
Seg	55.7		%	LDH	204	U/L
Ly	30.1		%	ALP	163	U/L
Mono	7.3		%	AMY	73	U/L
Eo	6.6		%	BUN	11.0	mg/dL
RBC	3.45 × 10^6^		/μL	Cr	0.58	mg/dL
Hb	12.0		g/dL	Glu	162	mg/dL
Ht	34.2		%	TP	5.8	g/dL
Plt	128 × 10^3^		/μL	Alb	3.5	g/dL
Coagulation				Na	140	mEq/L
PT		98.7	%	K	3.3	mEq/L
PT‐INR		1.01		Cl	107	mEq/L
APTT		52.2	sec	Ca	7.9	mg/dL
Fibrinogen		372.6	mg/dL	CPK	93	U/L
D‐dimer		1.01	μg/mL	CK‐MB	21	ng/mL
Biochemistry				Troponin T	0.015	ng/mL
AST		33	U/L	NT‐pro BNP	579.8	pg/mL
ALT		13	U/L	CRP	3.02	mg/dL

Abbreviations: Alb, albumin; ALP, alkaline phosphatase; ALT, alanine aminotransferase; AMY, amylase; APTT, activated prothrombin time; AST, aspartate aminotransferase; BUN, blood urea nitrogen; Ca, calcium; CK‐MB, creatine kinase‐muscle/brain; Cl, chloride; CPK, creatine phosphokinase; Cr, creatinine; CRP, C‐reactive protein; Eo, eosinophils; Glu, glucose; Hb, hemoglobin; Ht, hematocrit; K, potassium; LDH, lactate dehydrogenase; Ly, lymphocytes; Mono, monocytes; Na, sodium; NT‐pro BNP, N‐terminal pro‐brain natriuretic peptide; Plt, platelets; PT, prothrombin time; PT‐INR, prothrombin time international normalized ratio; RBC, red blood cells; Seg, segmented neutrophils; T‐bil, total bilirubin; TP, total protein; WBC, white blood cells; γ‐GTP, γ‐glutamyl transferase.

**Figure 3:**
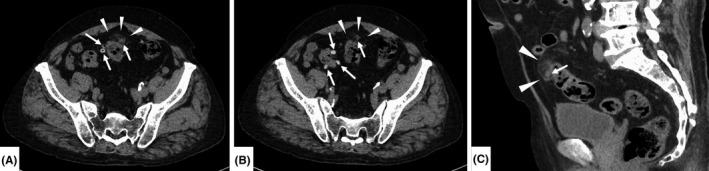
(A, B) Axial view. (C) Sagittal view. Thoracic and abdominal computed tomography without contrast enhancement shows multiple diverticula between the ascending colon and sigmoid colon (arrows). One of the diverticula near the sigmoid colon and adipose tissue around it show an area of high attenuation (arrowheads), suggesting a diagnosis of diverticulitis of the colon

Because of the presence of multiple risk factors for cardiovascular disease, serious and potentially fatal causes of chest pain—that is, the “five deadly causes of chest pain”—were initially considered to be the most likely diagnostic candidates. Thoracic and abdominal computed tomography (CT) were performed without contrast enhancement because of his history of allergic reactions to contrast material. The imaging, however, showed no abnormality in the thoracic region, especially in the vascular system. There were no findings suggesting the presence of esophageal cancer, pancreatic cancer, or cholecystitis, either. Only abnormalities revealed by CT were the presence of multiple diverticula between the ascending and sigmoid colon. A high CT value was observed in one of the diverticula in the sigmoid colon and adipose tissue around it, suggesting a diagnosis of colonic diverticulitis (Figure 3). Upper gastrointestinal endoscopy was not performed, because we did not suspect gastric mucosal disorders due to the lack of pain or tenderness in his epigastric region. Pulmonary embolism was ruled out due to the lack of meaningful elevation of D‐dimer with normal findings of electrocardiogram and echocardiography.

The patient was admitted to our hospital and treated conservatively under meticulous observation for his chest pain and myocardial abnormalities. Acute coronary syndrome was ruled out after confirming the absence of any changes in his ECG findings or cardiac enzyme titers 6 hours after their first evaluations. In addition, 24‐hour ECG monitoring for 6 days after his admission indicated no findings of changes in ST‐T segments or appearance of atrial fibrillation. Sigmoid colon diverticulitis was treated with 3 days of fasting in addition to 6 days of cefmetazole (3 g/day) administration.

His chest pain had disappeared by day 6 after admission, and his abdominal tenderness was diminishing gradually without deterioration. His chronic heart failure was stable and required no intervention. He was therefore discharged home on day 6, with the inflammatory reaction and symptoms having been alleviated with conservative treatment. CT with contrast enhancement fortuitously taken for another indispensable reason 7 months later showed the disappearance of high CT value of the diverticula and adipose tissue around it, without indicating the presence of any malignancies, pulmonary embolism, or abdominal arterial diseases causing intestinal ischemia including mesenterial thrombosis.

## 3 DISCUSSION

The patient in the present case was diagnosed with sigmoid colon diverticulitis, although he complained only of lower anterior chest pain before being admitted to our hospital. A previous study reported that 8.2% of patients who see a general physician because of chest pain are diagnosed with a digestive disease, like our patient, although the most common diagnoses were cardiovascular diseases, which accounted for 16.1%.^7^ In that study, among the 55 patients with digestive diseases who complained of chest pain, 42 were diagnosed with esophagitis, 5 with gastritis or a gastric ulcer, 5 with esophageal spasm, and 1 each with esophageal cancer, pancreatic cancer, and acute cholecystitis. None of them had a colonic disease.^7^ It is also reported that gastric or esophageal diseases often cause chest pain, as do gallbladder and pancreatic diseases.^8^ We had reported a patient with acute cholecystitis whose only complaint was chest pain—not abdominal pain—as an initial symptom.^1^ There have been no reports, however, of colonic diverticulitis presenting with chest pain alone and no spontaneous abdominal pain.

Referred pain—pain perceived at an area remote from the original site of the stimulus—is considered the mechanism of the lower anterior chest pain in the present case.^9^ Referred pain occurs when activation of nociceptors in the viscera results in perception of pain localized to the body surface at the same level of the spinal dermatome as that of the spinal cord of activated nociceptors. It occurs via a convergence of information from multiple nociceptor afferents on individual spinothalamic tract neurons.^10^ Although pain from the colon is transmitted via the Th10‐L1 nerve roots through celiac and adrenal plexuses, the referred pain could be perceived at the lower anterior chest because of the wide range of spinal roots below Th4 that join the celiac and adrenal plexuses.^11^ Therefore, the dermatome Th5‐6, where the chest pain of our patient was located, may be compatible with the site of the pain referred from the sigmoid colon diverticulitis. Arriving at a correct diagnosis for a patient presenting only with referred pain is challenging because the site of the pain does not directly indicate the location of the organ involved.^10^ When the physical examination and screening tests targeting the site of the pain fail to indicate the causative organ transmitting the pain, it becomes mandatory to consider the possibility of referred pain. To test this possibility, further appropriate imaging tests (eg, ultrasonography, thoracic and abdominal CT) are applied.^9^ In addition to the patients in our previous reports on referred pain,^1^, ^2^ several other patients presenting with pain at an unusual site have been reported. They include a fatal case in which myocardial infarction is presented as a headache ^12^ and an ectopic pregnancy that is presented as chest pain.^13^ The patient with the ectopic pregnancy was initially suspected to have a pulmonary embolism because of her chest pain. Abdominal CT showed the presence of ascites in the upper abdomen, leading to the correct diagnosis.^13^ It is thus essential to consider the possibility of referred pain when seeing a patient with pain, the site of which does not directly indicate the organ involved. One must not obsess about the site of the pain, which could delay making a quick, correct diagnosis.

Our patient with sigmoid colon diverticulitis, who presented with chest pain alone, though rare, indicates the importance of careful abdominal examinations for patients complaining only of chest pain who are suspected of having referred pain, after ruling out diseases that must be addressed with extreme urgency, such as the five deadly causes of chest pain.

## CONFLICT OF INTEREST

None declared.

## AUTHOR CONTRIBUTIONS

Contributors MT: involved in literature search, concept, and drafting. RH: involved in literature search, drafting, and clinical care of the patient. HY: involved in literature search and clinical care of the patient. SO: involved in literature search and clinical care of the patient. YT: involved in literature search and clinical care of the patient. HA: involved in literature search and drafting. MF: involved in literature search and drafting. SY: involved in concept and revision of article.

## ETHICAL APPROVAL

The patient gave permission for the publication of this case report. This manuscript conforms to the provisions of the Declaration of Helsinki in 1995 (as revised in Brazil 2013).

## Data Availability

All data underlying the results are available as part of the article, and no additional source data are required.
